# Langerhans cell histiocytosis manifesting at birth: a neonatal case with BRAF V600E mutation

**DOI:** 10.1186/s13000-025-01737-9

**Published:** 2025-12-30

**Authors:** Wenyan Tang, Ping Wang

**Affiliations:** Department of Neonatology, Guangzhou Women and Children’s Medical Center, Guangzhou Medical University, Guangzhou, Guangdong 510627 China

**Keywords:** Langerhans cell histiocytosis, Neonatal onset, BRAF V600E mutation, Chemotherapy, Long-term follow-up

## Abstract

**Background:**

Langerhans cell histiocytosis (LCH) is a rare histiocytic disorder characterized by clonal proliferation of abnormal Langerhans cells. We report a case of neonatal LCH diagnosed shortly after birth, an exceptionally early presentation in the neonatal period that is exceedingly rare. The BRAF V600E mutation was detected in this neonate.

**Case presentation:**

We report a full-term male neonate delivered via forceps assistance. Within 24 hours of birth, a firm 1×1 cm subcutaneous nodule on the left medial thigh progressively enlarged and ulcerated. By postnatal day 19, a dark red non-blanchable papule appeared on the left sole and rapidly spread to postauricular, cervical, truncal, and palmar regions, developing multiple ulcerative lesions. Skin biopsy confirmed Langerhans cell histiocytosis (LCH), with immunohistochemistry demonstrating diagnostic markers CD1a(+), Langerin(+), and S-100(+). Molecular testing detected the BRAF V600E mutation. Based on the early onset of the disease, rapidly progressive multifocal ulcerative skin lesions, and the presence of a high-risk BRAF mutation suggesting potential systemic dissemination, we initiated induction chemotherapy with vincristine combined with prednisone. Following treatment, the skin lesions resolved completely. The child is now 30 months old. During follow-up, an episode of otitis media occurred, but no recurrence or systemic organ involvement has been observed since.

**Conclusion:**

In this neonate, the initial localized skin lesions suggested potential spontaneous resolution. However, subsequent detection of the poor-prognosis BRAF V600E mutation indicated risk of systemic dissemination, prompting initiation of combination chemotherapy. Skin lesions resolved completely following vinblastine/prednisone therapy. Otitis media (an extracutaneous manifestation) emerging during follow-up further validated the treatment necessity, with no recurrence or new systemic manifestations observed thereafter.

## Background

Langerhans cell histiocytosis(LDH) is a rare histiobytic disorder characterized by clonal proliferation of abnormal dendritic cells, exhibiting significant clinical heterogeneity ranging from self-resolving skin lesions to life-threatening disseminated forms involving multiple organ systems [1, 2]. Although LCH predominantly affects children aged 1–3 years [3], neonatal LCH, which occurs in 1–2 per 1,000,000 newborns, manifests across the full disease spectrum; multisystem (MS) involvement is reported to be the most common presentation [4]; notably, presentation within 24 h of birth represents a rare clinical subset. The BRAF V600E mutation, a key molecular marker present in LDH patients [5], is strongly associated with increased risk of systemic dissemination and treatment resistance. Moreover, BRAF-V600E expression in precursor versus differentiated dendritic cells defines clinically distinct LCH risk groups [6]. BRAF V600E in bone marrow (without histologic infiltration) may indicate occult high-risk disease in neonatal limited cutaneous LCH; however, its prognostic significance is unknown.

Although most neonatal LCH cases initially present with isolated skin involvement, there is potential for future multisystem relapse [7], which makes long-term follow-up essential, especially in cases with rapidly progressive ulcerative lesions [8]. Systemic chemotherapy is often considered for all patients with MS-LCH [9]. However, how best to manage BRAF V600E-positive limited cutaneous LCH without systemic involvement is still debated.

This report presents a case of Langerhans cell histiocytosis (LCH) in a newborn who developed skin lesions within 24 h of birth. The lesions presented as ulcerated skin nodules and papules with an early onset (within 24 h postpartum). BRAF V600E mutation was detected in the bone marrow without evidence of tissue infiltration. The patient achieved complete remission following systemic chemotherapy. This case provides significant clinical implications for prognostic assessment and individualized treatment decisions in patients with skin-limited LCH positive for the BRAF V600E mutation in the bone marrow.

## Case presentation

### History of present illness

**Fig. 1:**
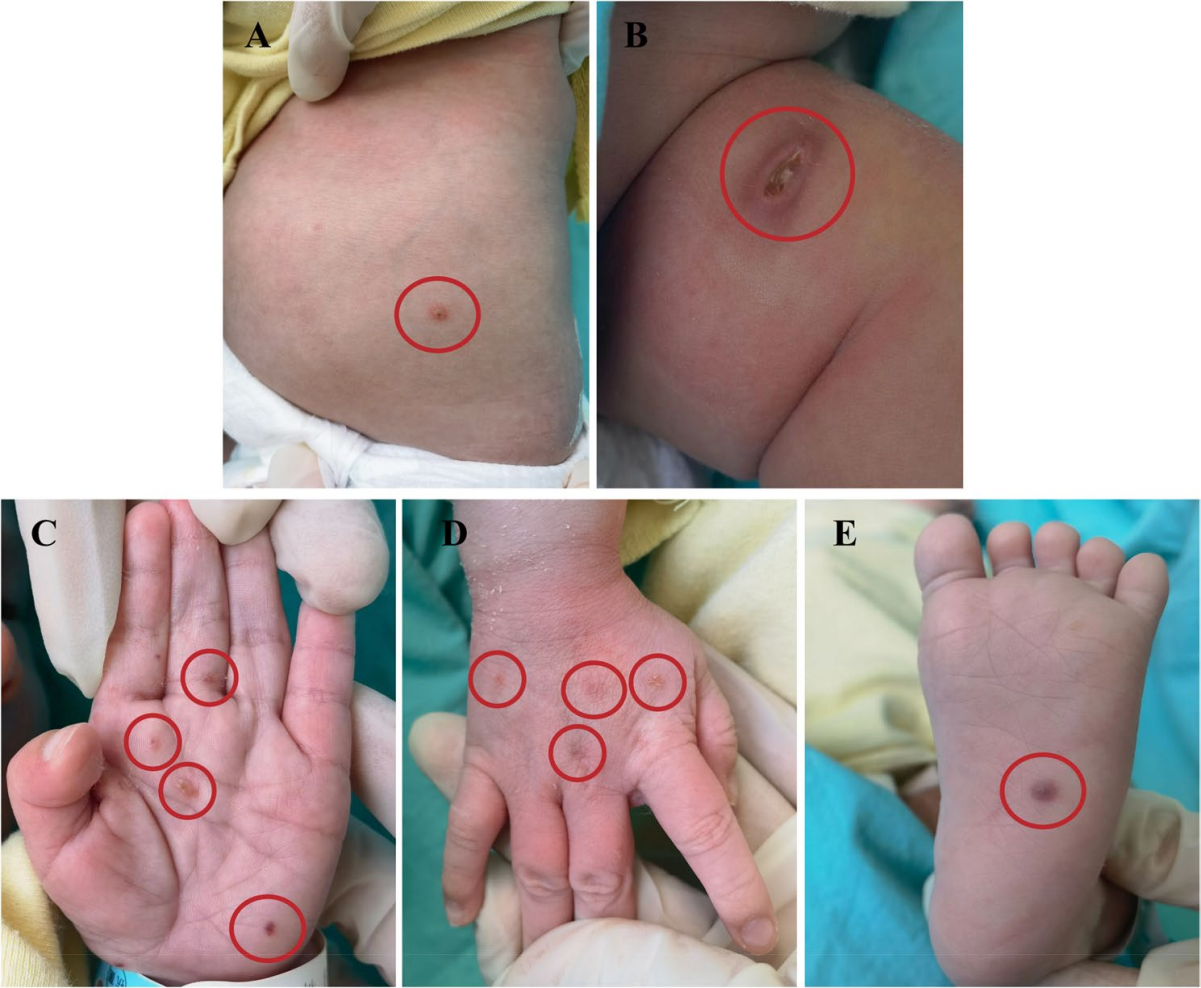
Cutaneous manifestations of Langerhans cell histiocytosis. **A **Abdominal rash, (**B**) thigh lesion, (**C**) central palmar involvement, (**D**) dorsal hand lesion, (**E**) diffuse palmar rash

A full-term male neonate was born at 39 + 1 weeks of gestation via forceps-assisted delivery without perinatal asphyxia. At birth, a 1 × 1 cm firm, non-tender subcutaneous nodule with ill-defined borders was noted on the left medial thigh. This lesion gradually enlarged and subsequently ulcerated. On postnatal day 19, a dark red, pea-sized, non-blanching, raised skin lesion appeared on the left sole. Subsequently, similar lesions developed on the posterior auricular area, neck, chest, abdomen, back, and palms (Fig. 1). Some lesions ulcerated. Topical treatments including mupirocin ointment, Kangfuxin solution, povidone-iodine, and recombinant human epidermal growth factor were applied without significant improvement. The neonate remained afebrile with no respiratory distress (no cough, sputum, cyanosis), gastrointestinal symptoms (no abdominal distension, diarrhea; normal stool), or urinary issues (adequate urine output).

### Physical examination

The neonate was alert and responsive. Multiple skin lesions, some ulcerated, were scattered over the body. No lymphadenopathy was palpable. The abdomen was slightly distended but soft, with normal bowel sounds. The liver and spleen were not palpable below the costal margin.

### Family history

 He has a healthy 4-year-old sister. There was no family history of genetic disorders, similar skin conditions, or other significant illnesses.

### Investigations

#### Routine labs

 Erology for TORCH (toxoplasmosis, rubella, cytomegalovirus, and herpes) group infections, Epstein-Barr virus, coxsackie virus A and B, adenovirus, parvovirus, and syphilis were negative. Complete blood count with CRP, biochemistry, and liver function tests were within normal limits.

#### Imaging

Skeletal survey (whole spine X-rays) and cranial ultrasound were normal except for a left subependymal cyst noted on cranial ultrasound.

Abdominal ultrasound (liver, gallbladder, spleen, pancreas, kidneys, ureters) was unremarkable.

Comprehensive radiological staging (including whole-body X-rays and computed tomography scans) revealed no evidence of extracutaneous organ involvement.

Pituitary MRI and urine osmolality were normal.

##### Bone marrow aspiration

Revealed hypercellular marrow. Molecular testing detected a BRAF V600E mutation.

##### Skin biopsy (lesion)

**Fig. 2:**
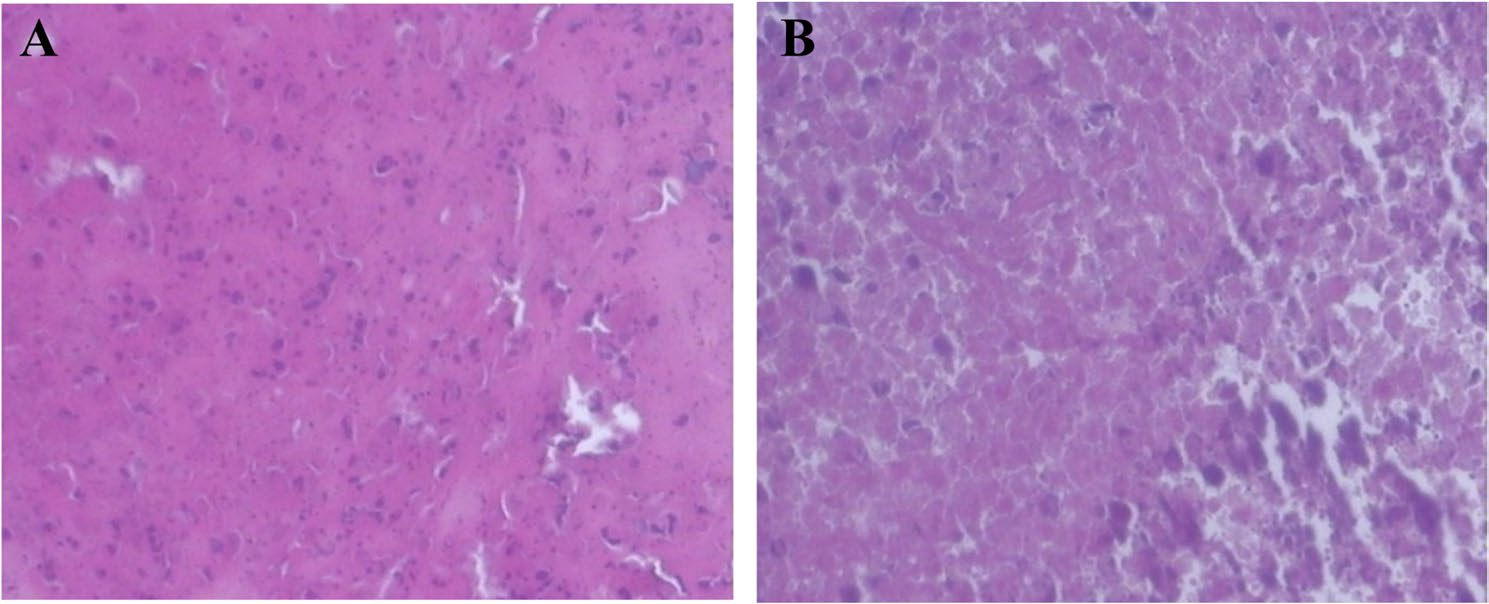
Histiocytic cells with pale cytoplasm and reniform nuclei on hematoxylin and eosin (H&E) staining, diagnostic of LCH. **A** x100; (**B**) x200

Hematoxylin and eosin (H&E) staining demonstrated histiocytic cells with pale cytoplasm and reniform nuclei, consistent with Langerhans cell histiocytosis (LCH) (Fig. 2).

##### Immunohistochemistry (IHC)

**Fig. 3:**
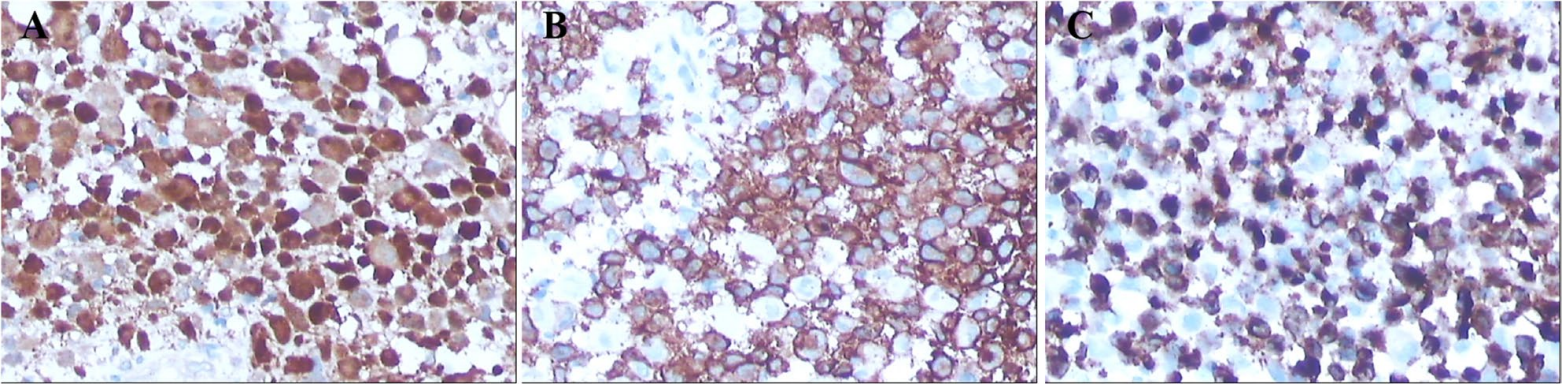
Immunohistochemical staining of lesional cells showing strong positivity for (**A**) S-100, (**B**) CD1a, and (**C**) Langerin (CD207)

The lesional cells were strongly positive for S-100, CD1a and Langerin (CD207) (Fig. 3).

#### Diagnosis

Based on the characteristic clinical presentation, histopathological findings (H&E), and confirmatory IHC profile (S-100+, CD1a+, Langerin+), a diagnosis of Langerhans cell histiocytosis (LCH) was established.

#### Treatment and course

Given the extensive multifocal cutaneous involvement and the detection of the BRAF V600E mutation (a finding of prognostic significance), the decision was made to initiate systemic chemotherapy. The neonate received induction chemotherapy with vincristine and prednisone, resulting in the gradual resolution of all skin lesions. The patient is currently 30 months old and receiving maintenance chemotherapy. During follow-up, a single episode of otitis media occurred; however, it did not recur, and no new lesions in other organ systems have developed.

## Discussion

Langerhans Cell Histiocytosis (LCH) is a group of rare histiocytic disorders arising from abnormal clonal expansion of bone marrow precursor cells. These precursors further differentiate into CD1a+/CD207 + cells, leading to varying degrees of organ involvement or dysfunction [1]. LCH is primarily classified into four clinical types: eosinophilic granuloma(EG), Letterer-Siwe syndrome(LSS), Hand-Schüller-Christian syndrome(HSC), and Hashimoto-Pritzker disease(HPD; also termed congenital self-healing LCH, CSHLCH). Of these, CSHLCH represents a distinct entity characterized by its exclusive cutaneous involvement and spontaneous regression tendency. Although lesions usually resolve spontaneously within months, the rash manifests as reddish-brown papules or nodules that may ulcerate, become necrotic, and then crust. This presentation is easily confused with neonatal hemangiomas, the “blueberry muffin” rash, or other rash-producing conditions. However, studies have found that even self-healing, solitary CSHLCH lesions can progress to widely disseminated multisystem LCH, therefore, long-term follow-up for evidence of relapse or progression of disease is essential [10].

The primary genetic susceptibility factors in LCH involve gain-of-function mutations in the mitogen-activated protein kinase (MAPK) signaling pathway, notably the BRAF V600E and MAP2K1 mutations [12]. The abnormal activation of the MAPK pathway, particularly its key kinase BRAF, plays a central role in the pathogenesis and progression of LCH [13]. Among its alterations, the BRAF V600E mutation represents the most common driver genetic alteration, leading to constitutive activation of the RAS/RAF/MEK/ERK signaling cascade, which drives abnormal cell proliferation and differentiation. Overexpression of this mutation in bone marrow progenitor cells recapitulates the LCH phenotype. By inducing senescence in multipotent hematopoietic progenitor cells (HPCs) and their secretion of senescence-associated secretory phenotype (SASP) factors, it promotes HPC differentiation bias towards the mononuclear phagocyte (MNP) lineage, ultimately resulting in the accumulation of senescent MNPs and the formation of LCH lesions [14]. Clinical studies demonstrate that the BRAF V600E mutation is a key biomarker predicting disease progression, relapse, and poor prognosis, and is strongly associated with multisystem involvement, particularly of high-risk organs such as the hematopoietic system, liver, and spleen [16]. The cell stage in which the mutation occurs determines the disease risk category: mutations occurring in early bone marrow precursor cells can progress to high-risk LCH, while mutations in differentiated mature dendritic cells lead to low-risk LCH [6]. This mutation is also significantly associated with high-risk disease features, permanent sequelae (e.g., neurological and pituitary damage), and poor short-term response to chemotherapy [6]. Multiple case reports confirm that children harboring the BRAF V600E mutation often present with multisystem/disseminated disease, exhibit chemotherapy resistance, and have a poor prognosis [17–19]. Therefore, in terms of treatment, BRAF V600E serves not only as a crucial predictor of disease progression but also as a potential therapeutic target for children with relapsed or multisystem disease [20]. Studies report that BRAF V600E-positive LCH responds poorly to conventional chemotherapy but might be sensitive to targeted inhibitors [11]. Consequently, it is recommended that children with LCH harboring this mutation receive BRAF-targeted inhibitor therapy. In contrast, children with cutaneous single-system LCH who are BRAF mutation-negative typically have disease confined to the skin, exhibit a favorable prognosis, and may undergo spontaneous resolution [15].

Although this neonate presented with skin-limited disease, molecular profiling revealed a BRAF V600E mutation. This finding implies that the mutation likely arose in an early myeloid progenitor cell with multilineage differentiation potential, thereby conferring inherent systemic dissemination capacity through myeloid pathways. Such molecular characteristics substantially elevate the risk of progression to multisystem high-risk LCH (involving liver, spleen, or bone marrow) and portend poorer outcomes. Consequently, based on the aggressive molecular signature, systemic induction chemotherapy (vincristine + prednisone) was immediately initiated. The neonate responded favorably with gradual resolution of generalized rash. At 30 months of age, the child remains on maintenance chemotherapy without recurrence of skin lesions.

## Conclusions

This neonate presented with progressive cutaneous eruptions on the extremities and abdomen during the neonatal period, requiring differentiation from infantile hemangiomas (lacking epidermal denudation) and infectious rashes (typically accompanied by elevated systemic inflammatory markers). Early and accurate recognition is critical to avoid misdiagnosis.The pivotal finding was detection of the BRAF V600E mutation in the bone marrow specimen. This molecular signature substantially elevates the risk of progression to multisystem high-risk LCH involving risk organs (liver, spleen, bone marrow). Consequently, we immediately initiated induction chemotherapy with vincristine + prednisone. At 30 months of age, the patient exhibits no disease recurrence or new systemic manifestations. We contend that limited LCH with bone marrow BRAF V600E mutation (even when classified as single-system skin disease) should be regarded as a potential high-risk subtype, warranting aggressive systemic therapy and long-term intensive surveillance to evaluate definitive outcomes.

## Data Availability

No datasets were generated or analysed during the current study.
